# Kartagener’s syndrome: review of a case series

**DOI:** 10.1186/s40248-015-0015-2

**Published:** 2015-05-30

**Authors:** Nicola Ciancio, Maria Margherita de Santi, Raffaele Campisi, Laura Amato, Giuseppina Di Martino, Giuseppe Di Maria

**Affiliations:** Pulmonology Unit, A.O.U. Policlinico-Vittorio Emanuele, Catania, Italy; Department of Human Pathology and Oncology (Division of Pathology), University of Siena, Siena, Italy; Department of Clinical and Molecular Biomedicine, University of Catania, Catania, Italy

**Keywords:** Dinein arms, Kartagener’s syndome, Nasal brushing, Primary ciliary dyskinesia, *Situs inversus*, Transmission electron microscopy

## Abstract

**Background:**

Kartagener Syndrome (KS) is a rare autosomal recessive genetic disorder, resulting in a group of clinical manifestations, including bronchiectasis, chronic pansinusitis and situs inversus.

**Methods:**

We hereby reviewed eight cases of this rare entity selected from patients attending our outpatients Respiratory Unit since 2006. Samples of respiratory epithelium were obtained with the method of nasal brushing and sent to a specialized center in order to be studied with electron microscopy. At least 50 cross sections of different cilia from different cells were observed in each specimen to study the axonemal structure. Electron micrographs were taken at a magnification of X 50,000 to determine the orientation of the cilia and at a magnification of X 110,000 to study the axonemal pattern. The incidence of abnormal cilia was expressed as a percentage.

**Results:**

We observed different ultrastructural defects in our KS patients, including absence of outer dynein arms, absence of outer and inner dynein arms, and absence of the central pair with transposition of a peripheral doublet into the central position. Patient’s follow up lasted till 2014, however two patients with more severe clinical behavior died before.

**Conclusions:**

This is a review of a case series, yet our data has shown that nasal brushing with ultrastructural pathological differentiation may be useful to identify patients with high risk and to develop more complex clinical presentations.

## Review

### Introduction

Primary Ciliary Dyskinesia (PCD) is a genetically and phenotypically heterogeneous hereditary disorder mainly transmitted by autosomal recessive inheritance. The genetic basis of the variety of defects affecting ciliary structure and function in PCD is not clear: to date, mutations in more than 30 different genes have been referred [1]. The disease occurs as a direct result of congenital defects in motile cilia covering the respiratory epithelia, leading to impairment of the mucociliary clearance. PCD is characterized by chronic upper and lower respiratory tract infections; the clinical phenotype is broad and overlaps with other chronic airways diseases; the incidence and the severity differs from one patient to another, even among siblings. The estimated prevalence of PCD is about 1 in 16,000, but this could be an underestimation due to missed diagnosis. Around 50% of the patients with PCD have a mirror image arrangement of their internal organs. The triad of mirror image arrangement, bronchiectasis and chronic sinusitis is known as Kartagener syndrome (KS). Inversion of *situs* in PCD is a random event as proved with monozygotic twins with discordant heart orientation [2]. Cilia rotation induces a leftward flow to the extraembryonic fluid. This flow may concentrate on the left side, or deplete on the right side, the critical factors that start the molecular cascade needed for normal lateralization [3]. If the flow is not present, the factors are equally distributed and the lateralization is randomized. Although Siewert first described this condition in 1904, Kartagener just recognized the etiological correlation between the elements of the triad and reported four cases in 1933 [4,5]. As extrapolated from radiographic studies, the incidence of KS is estimated at 1/32,000 births based on the prevalence of situs *inversus* and bronchiectasis [6]. In the 1970s, Bjorn Afzelius, reported cilia immobility in infertile males, some of the cases occurring in families [7]. Half of the cases had Kartagener’s triad. For this reason it would be appropriate to call the condition Kartagener-Afzelius syndrome. Subsequently, with the introduction of electron microscopy studies, these patients were noted to have immotile cilia and defects in the ultrastructural organization of cilia [8,9]. Initially, the term immotile cilia syndrome was used to describe this disorder; however, later studies showed that most cilia were motile, but exhibited a stiff, uncoordinated, and/or ineffective beat. The name was changed to “PCD” to more appropriately describe the heterogeneous genetic base and the ciliary dysfunction and to distinguish it from the secondary ciliary defects acquired after multiple causes of epithelial injury [10, 11]. These defects could affect the ciliary movements of the respiratory epithelium, determining recurrent and/or persistent sinopulmonary infections. Cilia dysfunction is also implicated in a wider spectrum of disease, including polycystic liver and kidney disease, central nervous system problems including retinopathy and hydrocephalus, and biliary atresia [12-14]. Establishment of diagnosis currently relies on a combination of clinical evaluation and electron microscopy examination of defective ciliary ultrastructure, even though a 3-30% of patients with clinical features of PCD are reported to display normal cilia, which further confounds the diagnosis [15]. The saccharin method for testing mucociliary function and electron microscopy of respiratory cilia, obtained by nasal scrape or brush biopsy complete the diagnostic workup of the patients [16,17]. A few specialized centers use high-speed videomicroscopy to examine ciliary beat. Certain beat patterns correlate with ultrastructural defects, and, in some cases, subtle alterations in beat pattern can be seen when ultrastructure is normal [18]. Raidt and coll, describe a large cohort of patients with defined mutations in multiple PCD genes and the associated abnormalities of ciliary pattern observed by high-speed video-microscopy analysis (HVMA), using freshly obtained nasal epithelium. This study confirms previous observations, showing that certain patterns of ciliary dyskinesia apparent on HVMA are associated with specific ultrastructural defects [19]. Recent studies have shown that nasal nitric oxide (NO) is very low in patients with KS compared with healthy control subjects; therefore, this assay may be a useful screening or adjunctive test for KS/PCD [20,21]. Because acute respiratory illnesses may yield alterations in ciliary ultrastructure, ciliary beat, and nasal NO values, these tests should be performed during a stable baseline period. Understanding the genetic basis of PCD has increased exponentially since when, in 1999, mutations in DNAI1 were reported to cause PCD [22]. To date, mutations in over 30 PCD-associated genes have been identified, accounting for > 60% of PCD cases [1,23].

In conclusion, research into ciliopathies has rapidly expanded, involving multidisciplinary efforts to define the complex genetics, clinic and functional phenotypes of cilia. We reviewed eight cases of KS in patients attending our outpatients Respiratory Unit since 2006.

## Patients and methods

We studied 8 consecutive KS patients (4 M and 4 F), attending to our department since 2006. Patients were recruited over a period of 24 months, when a sensitization campaign of awareness, regarding KS, was launched in our region among General Practitioners. Subject’s age is referred to the time of first visit to our clinic. Out of patients 2 died, 2 missed the follow up visits, and 4 are still in follow up in our outpatients laboratory.

### Transmission electron microscopy

For electron microscopic evaluation, it is important to optimize the size and the quality of the specimens, obtaining a sample comprised primarily of ciliated epithelial cells. This may be accomplished conveniently by sampling the nasal epithelium using a cytological brush or a disposable plastic curette designed for this specific purpose. It should be of importance to investigate at least two different mucosal sites because denudation or metaplasia of the nasal mucosa are rather common in these patients. Samples were obtained and treated with a standardized method [24]. Samples of respiratory epithelial cells obtained with the method of nasal brushing, were immediately fixed in 2.5% cacodylate-buffered glutaraldehyde pH 7.3 for 3 hours at 4 C°, washed overnight in the same buffer. To process the samples for electron microscopy and perform detailed ultrastructural analysis of the ciliary axoneme, the specimens were sent to a specialistic diagnostic center where they were postfixed in buffered 1% osmium tetroxide for 1 hour, washed, dehydrated through a graded series of ethanol, cleared in propylene-oxide and embedded in Epoxy resin (Araldite). Semithin sections 1 μm thick, cut with glass knives on an LKB V Ultrotome and stained with 1% toluidine blue, were examined with the light microscope in order to make an overall assessment of the tissue morphology. Ultrathin sections from selected areas were cut with a diamond knife using the same ultramicrotome, retrieved onto copper grids, double-stained with uranyl acetate and lead citrate and examined at 100 kV with a Philips 208 S transmission electron microscope. At least 50 cross sections of different cilia from different cells were observed in each specimen to study the axonemal structure. Only full cross-sectioned cilia were evaluated, excluding those near the base or tip. Electron micrographs were taken at a magnification of X 50,000 to determine the orientation of the cilia and at a magnification of X 110,000 to study the axonemal pattern. Dynein arms and microtubules were counted and the organization of the axoneme, the presence of radial spokes, and nexin links, spoke heads and central sheaths were evaluated. The incidence of abnormal cilia was expressed as a percentage. From each specimen, ciliary orientation was investigated observing at least 10 different cells. The ciliary axis was determined drawing a line through the central microtubular pair of each cilium. At least 10 suitable ciliary cross sections per cell were studied: the angles between the ciliary axis and a standard reference line were measured and the standard deviation of the angles per cell was calculated. Finally, the mean standard deviation of the examined cells of the same specimen was calculated.

## Case reports

### Case 1

**Figure 1:**
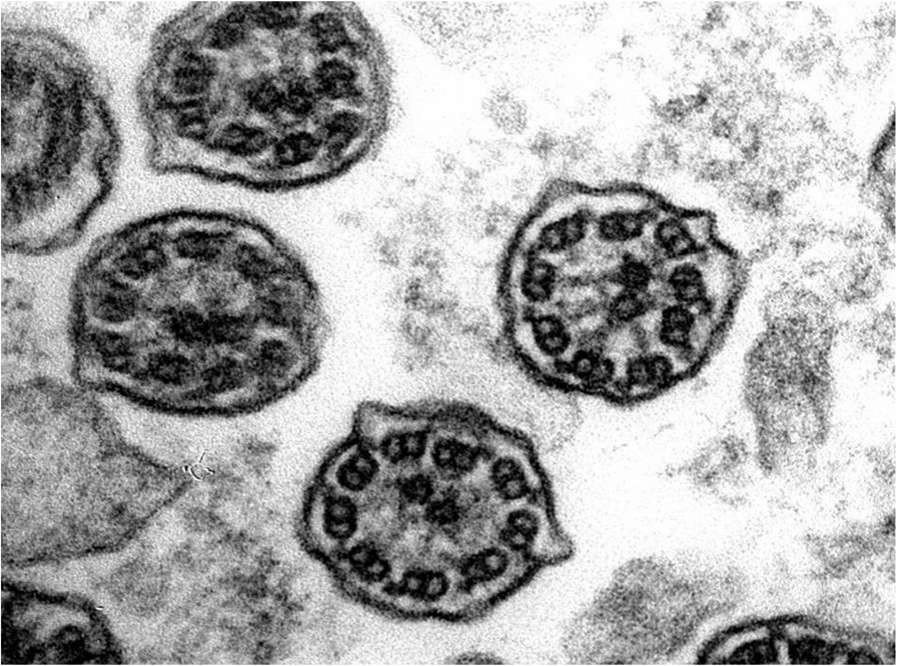
Transmission electron microscopy (EM) of representative nasal epithelium cilia from patient #1 with primary ciliary dyskinesia caused by demonstration of cilia lacking of inner dynein arms and ciliary abnormalities of symmetry presumably related to defects in the radial spokes.

This was a 30-year-old non-smoker male, born by non-consanguineous parents. He presented to the outpatient clinic (in May 2006) with chief complaints of recurrent episodes of common cold, sneezing, and cough with expectoration for the past 10 years, exertional shortness of breath for the last 5 years. The patient also revealed that he frequently developed cough, cold, rhinorrhea, nasal blockade, and ear discharge during childhood. At the time of the first visit, the vital parameters were within normal limits. Physical examination revealed grade 2 digital clubbing. On auscultation, bilateral wheeze and right basal crackles were audible. Electrocardiogram showed evidence of dextrocardia. Chest CT scan was done on May 18, 2006, and revealed dextrocardia, and diffuse bronchiectasis. Spirometric evaluation showed moderate obstruction. In the follow up visits the patients showed a progressive impairment of functional respiratory pattern. In 2007 the nasal brushing was performed, with demonstration of 100% cilia lacking of inner dynein arm associated with axonemal disorganization in 60% of the cilia, presumably related to defects in the radial spokes (Figure 1). In 2008 pulmonary function tests showed severe obstruction in spirometry and respiratory failure with low oxygen level in artery blood sample. The patient died in 2010.

### Case 2

**Figure 2:**
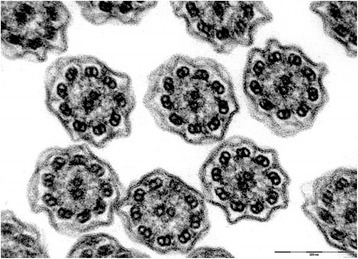
Transmission electron microscopy (EM) of representative nasal epithelium cilia from patient #2 with primary ciliary dyskinesia caused by demonstration of cilia lacking of lacking outer dynein arm.

This was a 39-year-old smoker female, born by non-consanguineous parents. Married with two children. She was hospitalized in August 2006 for acute exacerbation of bronchitis with cough and mucopurulent expectoration, and hyperthermia. She reported recurrent episodes of common cold, with productive cough and rhinorrhea since the childhood. Chest CT scan was done on August 31, 2006, and revealed dextrocardia, and bronchiectasis with prevalent diffusion in the right lung. On auscultation, right basal crackles were audible. Electrocardiogram showed evidence of dextrocardia. Spirometric evaluation showed moderate obstruction. Therapy with the DPI fixed combination formoterol/budesonide was established. In the follow up visits the patients showed a stabilization of functional respiratory pattern. In 2007 the nasal brushing was performed, with demonstration of 100% cilia lacking outer dynein arm (Figure 2). She missed the following up visits.

### Case 3

**Figure 3:**
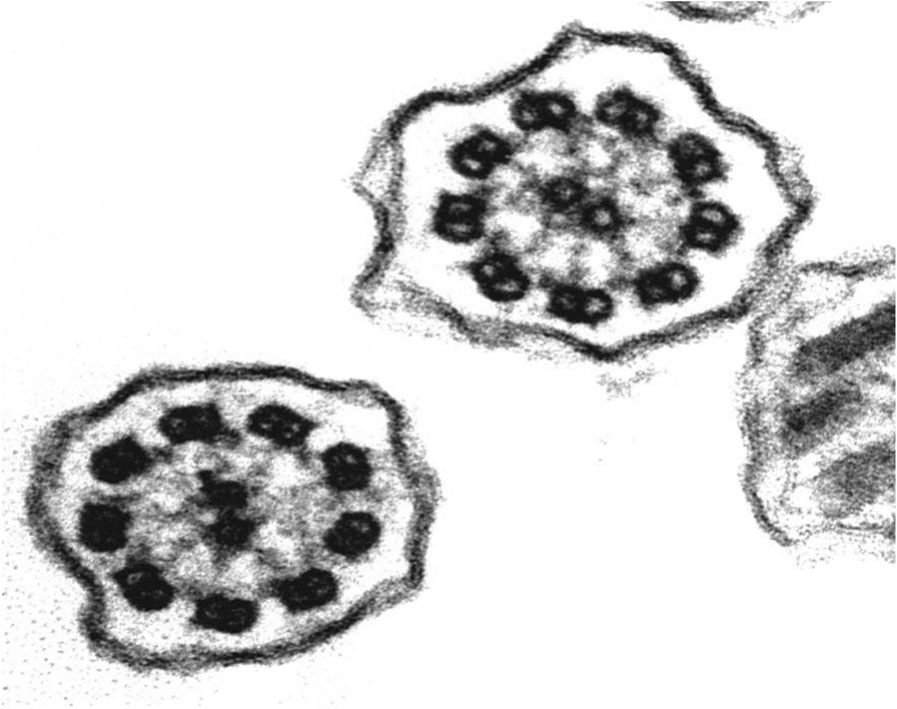
Transmission electron microscopy (EM) of representative nasal epithelium cilia from patient #3 with primary ciliary dyskinesia caused by demonstration of cilia lacking of lacking outer dynein arm.

**Figure 4:**
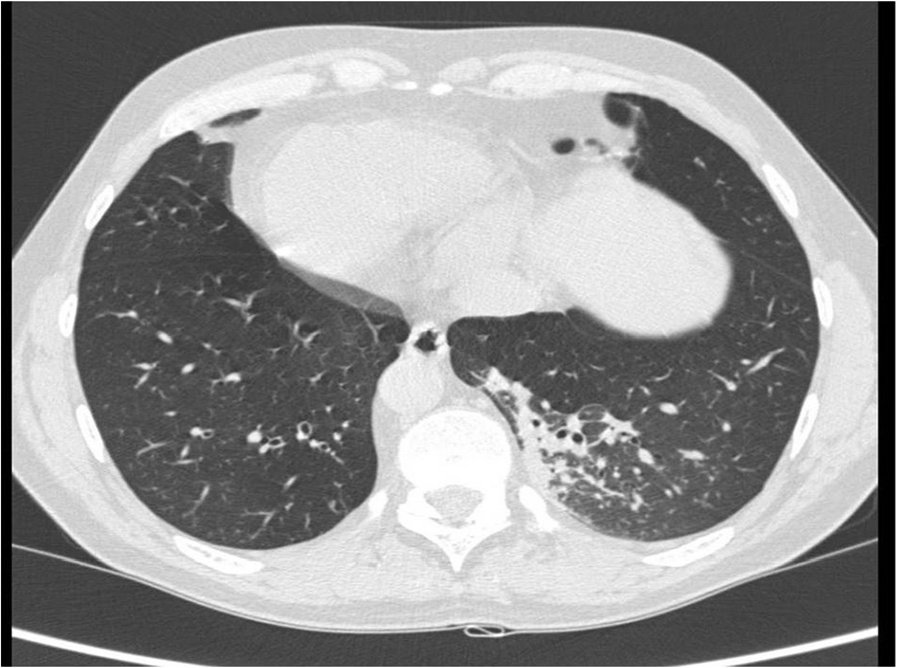
CT-scan performed in patient #3 during hospitalization for pneumonia in lower left lobe.

This was a 42-year-old non-smoker male, born by non-consanguineous parents. He presented to the outpatient clinic (in September 2006) with chief complaints of recurrent episodes of common cold, cough with expectoration for the past 15 years. The patient also revealed that he frequently complained of developed cough, cold, rhinorrhea, and wheezing since his childhood. At the time of the first visit, the vital parameters were within normal limits. On auscultation, bilateral wheezes without crackles were present. Electrocardiogram showed evidence of dextrocardia. At the time of the first visit, spirometric evaluation showed mild obstruction. Therapy with association salmeterol/fluticasone via DPI was established, with stabilization of functional respiratory pattern, in the follow up visits. In 2007 the nasal brushing was performed, showing all examined cilia lacking outer dynein arm (Figure 3). He was hospitalized for pneumonia to the lower left lobe as reported in the chest CT scan done on September 18, 2013 (Figure 4). The patient is still in follow up, in maintenance therapy and treated for exacerbations.

### Case 4

**Figure 5:**
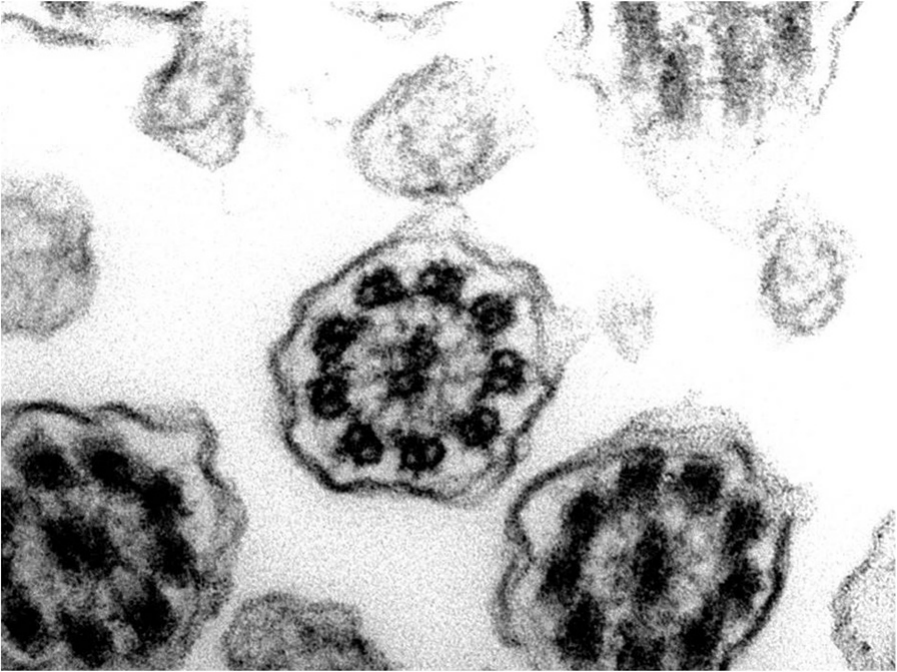
Transmission electron microscopy (EM) of representative nasal epithelium cilia from patient #4 with primary ciliary dyskinesia caused by demonstration of cilia lacking of lacking outer dynein arms.

**Figure 6:**
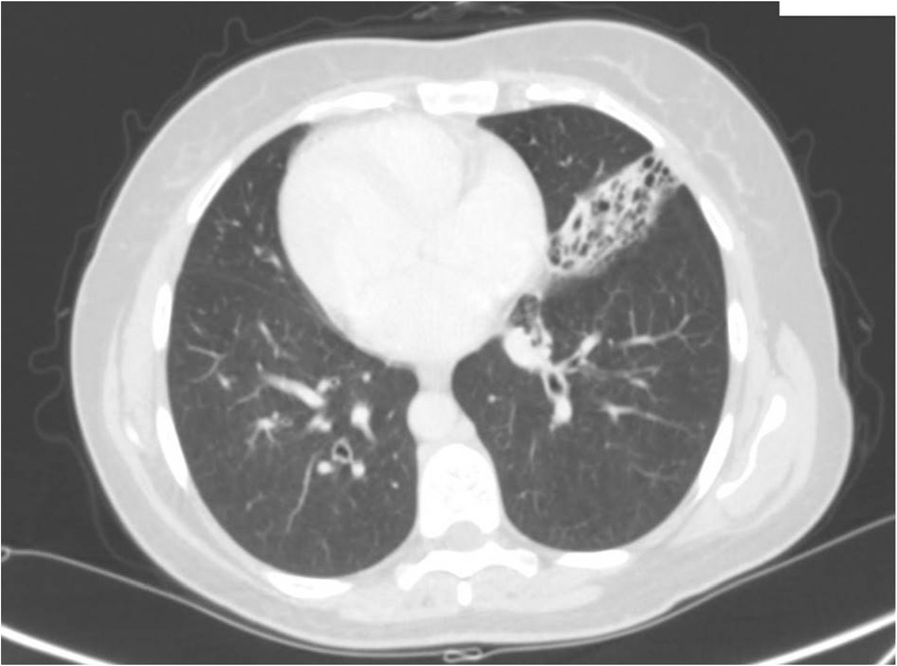
CT-scan performed in patient #4 during hospitalization for pneumonia in upper left lobe.

This was a 32-year-old non-smoker female. She presented to the outpatient clinic (in October 2007) with chief complaints of recurrent cold, cough with copious expectoration, and progressively increasing shortness of breath for the last 3 years. She had been married for the last 6 years, without children. Her past history was significant in that she had had frequent visits for recurrent chest infections. Her family history revealed no parental consanguinity. Main purpose of the visit in our Unit was a planned pregnancy. At the time of the first visit, the vital parameters were within regular limits, and thoracic auscultation was normal. The patients was fostered to pregnancy, and she has had three children so far. In 2008 the nasal brushing was performed, showing the absence of outer dynein arms in all the ciliary sections (Figure 5). In the last year, she suffered of some exacerbations and pneumonia, with 2 hospitalizations in our Unit. She had more chest CT scans, and the last one was done in 2013 (Figure 6). Spirometric evaluation at the follow up visits showed mild to moderate obstruction, and therapy with the MDI fixed combination formoterol/beclometasone was established, with stabilization of functional respiratory pattern between the crisis. Exacerbations were treated with antibiotics, and she is still in maintenance therapy with LABA/ICS inhalant therapy. In 2014 diagnosis of autoimmune thyroiditis was done.

### Case 5

**Figure 7:**
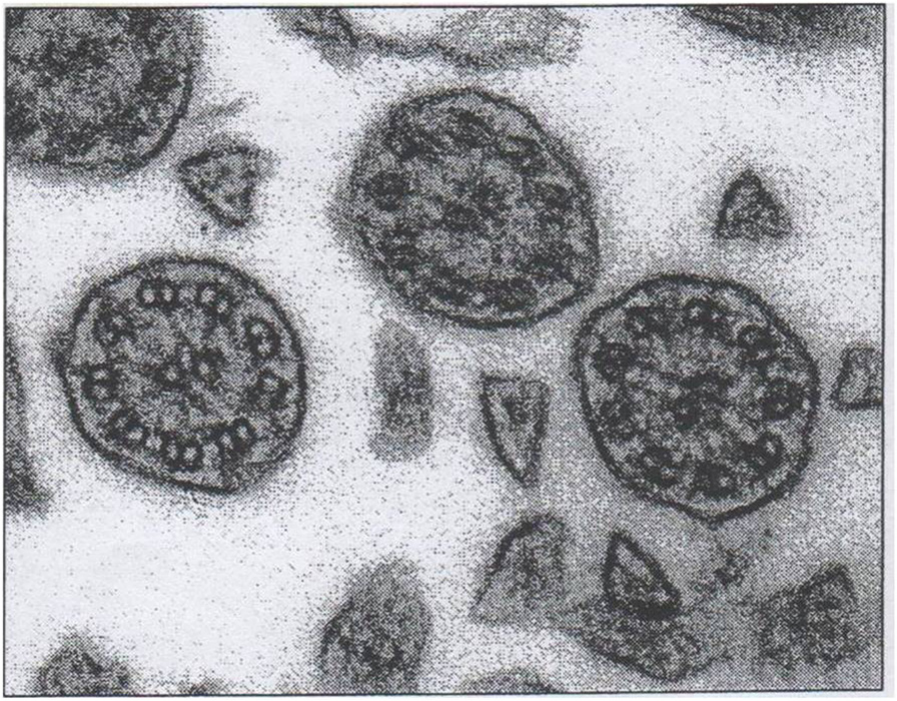
Transmission electron microscopy (EM) of representative nasal epithelium cilia from patient #5 with primary ciliary dyskinesia caused by demonstration of cilia lacking of lacking outer dynein arm.

This was a 62-year-old non-smoker female. Married with one child. She presented to the outpatient clinic (in October 2007) complaining of cough with abundant mucopurulent expectoration, many bronchitic exacerbations (> 3 episodes/yrs), recurrent cold, and progressively increasing breathlessness in the past 5 years. In her past history there were frequent visits for recurrent chest infections and pneumonia. At the time of the first visit, the vital parameters were within regular limits, whereas at thoracic auscultation bilateral crackles in the basal region were present. Basal spirometric evaluation showed moderate obstruction with positive reversibility test. Therapy with association of salmeterol/fluticasone was prescribed, with stabilization of respiratory values at the follow up visits. In 2008 the nasal brushing was performed, with demonstration of 100% of cilia lacking outer dynein arms (Figure 7). In the last years she suffered of 2/3 exacerbations per year, without need of hospitalization.

### Case 6

**Figure 8:**
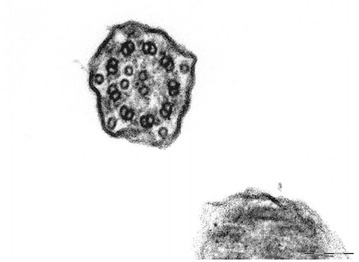
Transmission electron microscopy (EM) of representative nasal epithelium cilia from patient #6 with demonstration of cilia with presence of both dynein arms but abnormalities in number and disposition of outer microtubular pair.

**Figure 9:**
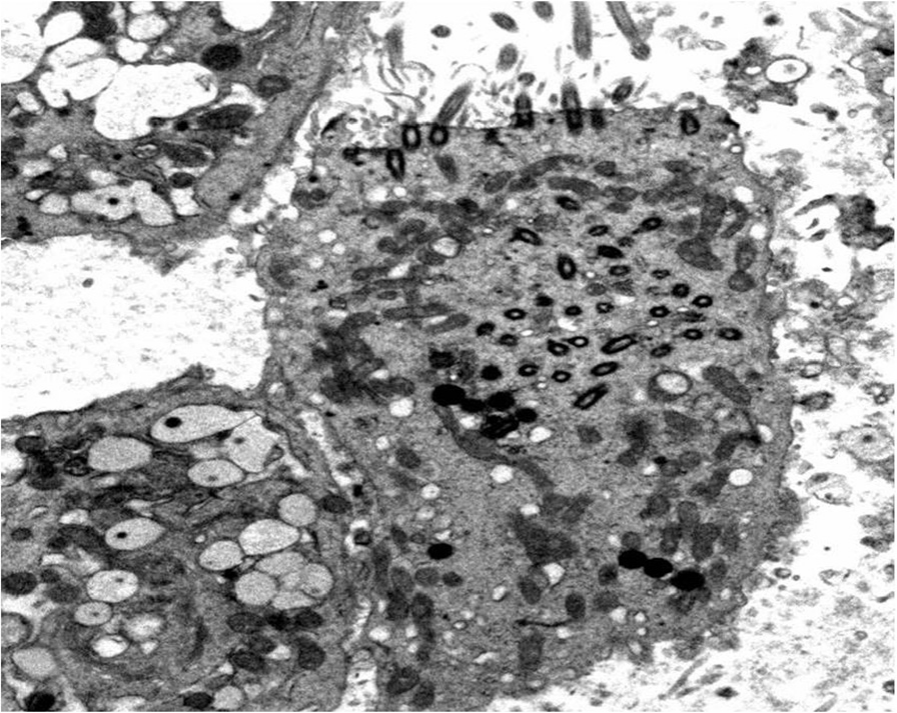
Patient #6, the cilia were asymmetric, some of them lacking of central pair. Basal bodies showed an atypical distribution.

This was a 43-year-old non-smoker male, born by non-consanguineous parents, who had a mental retardation due to perinatal hypoxemia. He had several hospitalizations in our and other pneumology units, for severe acute exacerbation of bronchitis, characterized by continuous cough with purulent sputum, fever, wheezing and dyspnea. He suffered from recurrent episodes of common cold, cough with expectoration and exertional dyspnea since his childhood. At the time of the first visit to our outpatients clinic in 2007, physical examination revealed grade 1 digital clubbing. On auscultation, bilateral wheeze and bilateral basal crackles were audible. Bacteriological examination of sputum showed *Acinetobacter Iwoffii* infection. Spirometric evaluation showed moderate obstruction. He was treated with antibiotics and association of salmeterol/fluticason for maintenance inhalation therapy. Nevertheless, he suffered from several exacerbations and needed some hospitalizations. Chest CT scan was done during one of this hospitalizations and revealed dextrocardia, diffuse bronchiectasis and widespread micronodularity. Transcutaneous O_2_-saturation was low (89% at rest), and long-term O_2_-therapy was prescribed along with aerosol therapy. In 2008 the nasal brushing was performed, showing all cilia with both dynein arms. About 60% of cilia displayed aberrant number and/or localization of central or peripheral microtubules (Figure 8). Basal bodies showed an atypical distribution (Figure 9). In the follow up visits the patients showed a progressive impairment of functional respiratory pattern. The patient died in 2012.

### Case 7

**Figure 10:**
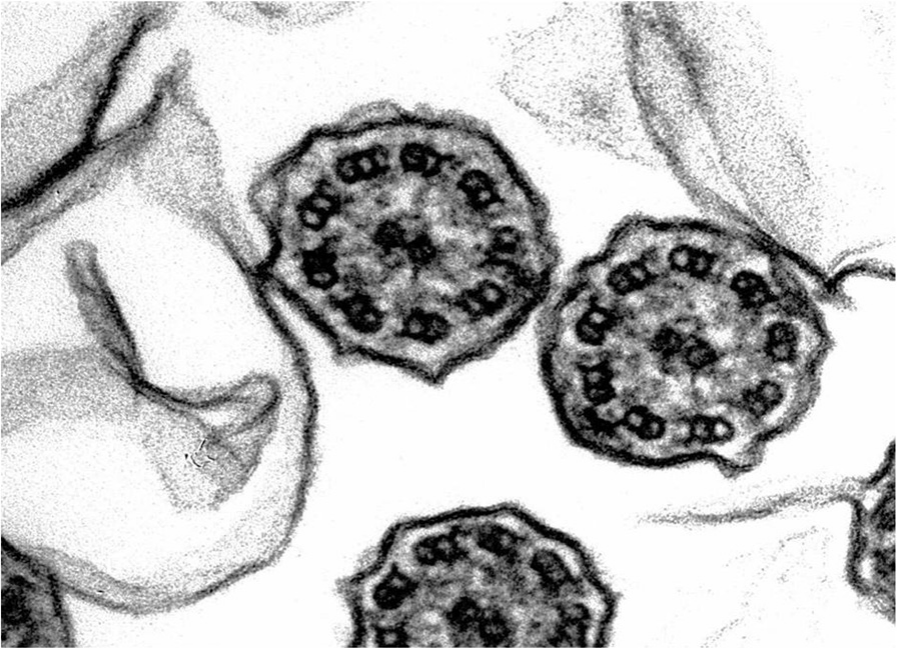
Transmission electron microscopy (EM) of representative nasal epithelium cilia from patients #7 with demonstration of 50% normal ciliated cells. In the remaining 50% ciliated cells axonemes lacking both dynein arms were found.

This was a 21-year-old non-smoker male, born by non-consanguineous parents. He presented to our outpatient clinic in December 2007, presenting personal CT-scan prescribed by his General Practitioner. Patient clinical history was characterized by recurrent cold, bronchitis and rhinorrhea since his childhood. At the time of the first visit, the vital parameters were within normal limits and thoracic auscultation was physiologic without wheezing and/or crackles. Electrocardiogram showed evidence of dextrocardia. Spirometric results were within normal range. No therapy was necessary. In 2008 the nasal brushing was performed, with demonstration of 50% normal ciliated cells. In the remaining 50% ciliated cells axonemes lacking both dynein arms were observed (Figure 10). The patient missed the follow up visits.

### Case 8

**Figure 11:**
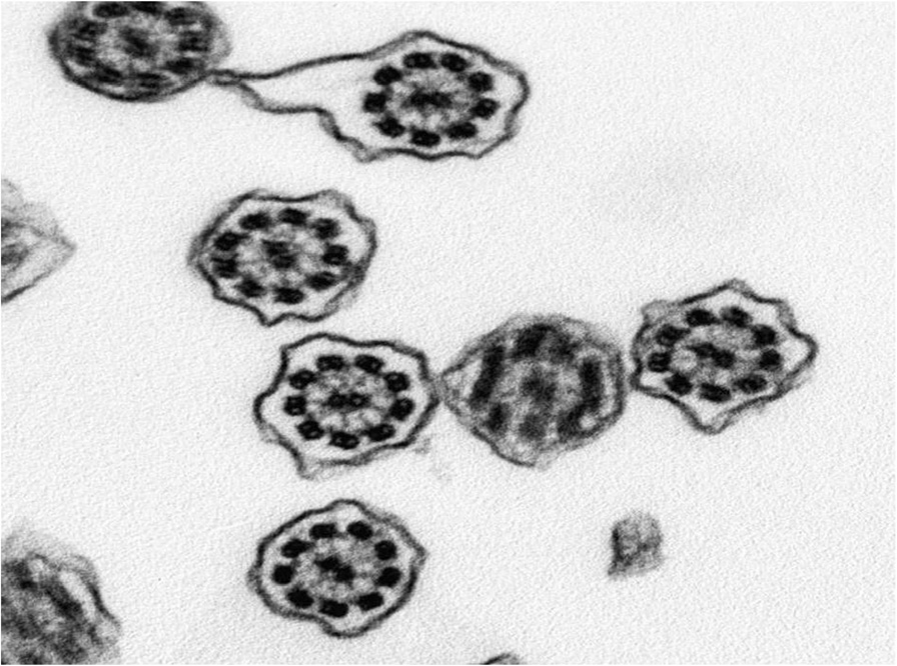
Transmission electron microscopy (EM) of representative nasal epithelium cilia from patients #8 with primary ciliary dyskinesia caused by lacking of inner dynein arms.

This was a 40-year-old non-smoker female, married with two children. She was hospitalized in 2005 in another public hospital in which thoracic CT-scan was performed with diagnosis of KS. She attended our outpatient clinic in 2008, for a routine control visit prompted by his General Practitioner. Patient’s clinical history was characterized by recurrent cold, bronchitis and rhinorrhea since her childhood. At the time of the first visit, the vital parameters were within normal limits and thoracic auscultation yielded a normal finding without wheezing and/or crackles. Spirometric exam was within normal range. No therapy was necessary. In 2008 the nasal brushing was performed, with 100% of the cilia lacking inner dynein arms (Figure 11). She suffered only few exacerbations (less than 1/yrs) without need of hospitalization. In the follow up visits respiratory function was stable and no bronchodilator therapy has been prescribed so far.

## Discussion

Bronchiectasis in KS patients, which develops after birth as an acquired condition, is defined as localized and irreversible dilatation of the part of the bronchial tree. The involved bronchi are dilated, inflamed and easily collapsible, resulting in airflow limitation, obstruction and impaired clearance of secretions, that can easily lead to respiratory infection, contributing to the common purulent expectoration observed in these patients. The result is a bronchial injury and a vicious cycle of bronchial damage, bronchial dilation, impaired clearance of secretions, recurrent infections, and further bronchial damage does establish. In clinical practice, this condition is most often characterized by coughing and daily production of mucopurulent sputum lasting from months to years. The classic symptoms triad of chronic cough, excessive production of purulent sputum, and repeated infections is seen in most of the patients. From the physiopathological point of view, a significant airway obstruction and airflow limitation may occur as consequence of the chronic bronchitis, bronchiolitis, and emphysema which are often associated with bronchiectasis. These physiopathological impairment could lead to the respiratory failure in patients with more severe clinical status. Pneumonia and chronic bronchial infection are common, and could contribute to deterioration of respiratory function. The third component of the KS triad is pansinusitis, an acquired condition which occurs in almost 100% of patients affected with this disorder. Once these patients present abnormal ciliary movements, there is an accumulation of secretions inside the paranasal sinuses. It could become a chronic process causing hypoplasia or even agenesis of the paranasal sinuses. Symptoms in KS-patients present from early childhood, and represented by frequent colds, nasal secretion, respiratory allergies, migraine and recurrent pneumonic infections. Treatment of KS is manly based on the prevention of repeated infections that might worsen bronchiectasis, resulting in a more severe airflow limitation and a more rapid decline of the lung function, together with a run-down condition due to possible severe pneumonia with risk of sepsis. Following diagnosis, the goals of respiratory management are the improvement of lung function and the limitation of disease progression. Treatment of lung disease is based upon airway clearance enhancement and aggressive antibiotic therapy [15]. Prophylaxis with appropriate measures of immunization, particularly with influence and pneumococcal vaccination, and strong pulmonary toilet are the mainstays of therapy [25]. Patients who develop recurrent pneumonia or haemoptysis and do not respond to antibiotics may benefit from segmental lung resection or lobectomy [26]. Monitoring the progression of lung disease in KS-PCD is difficult for many reasons. Decreased quality of life is caused by chronic respiratory symptoms and deterioration of respiratory function. For this reason, patients with KS-PCD should be regularly followed up by experienced specialists who have access to an array of monitoring tools in order to assess their airway disease, including pulse oximetry and appropriate lung function tests. However, there have been no long-term randomised trials of therapy in PCD, and there is a lack of evidence-based medicine in the management of this condition [15]. It is well known that diagnosis is frequently made late [12], partly because the disorder presents with symptoms (rhinitis, secretory otitis media, cough) which are common in children. Although there is no proven evidence that early diagnosis is beneficial, in one series of PCD patients, bronchiectasis at diagnosis was only seen in those diagnosed over 4 years of age [12], and in a second series, lung function at diagnosis was significantly worse in those diagnosed in adult life [27]. This is at least an evidence to support that early diagnosis is beneficial in PCD, and likely also in KS patients.

## Conclusions

In this review of eight case series, we observed two subjects who died in young age for respiratory failure (CASE 1 and 6). Transmission Electron Microscopy examination of the respiratory cilia showed the complete absence of both outer and inner dynein arms in case 1, whereas case 6 displayed cilia with the loss of inner dynein arms associated with disarranged axoneme and defective radial spokes. Despite these selected cases, the long-term prognosis of patients with KS is usually good, with many patients living till an advanced age, especially if patients and caregivers are trained in the proper management of the early signs and symptoms of a bronchitic exacerbation and possible respiratory infection.

Treatment of patients with KS is not well standardized, and many patients receive suboptimal management, including those who do not have regular surveillance of respiratory function, and many who have not received regular treatment long-life. On the other hand, from a diagnostic point of view, nasal brushing with ultrastructural pathological differentiation, till now not extensively used, is probably necessary to identify patients with high risk to develop more complex clinical presentations, as our data suggest. Thus, a multidisciplinary approach, including the contribute of Pulmonary, Radiology and Pathology specialists, is necessary to the appropriate diagnostic pathway and management of this particularly rare disease.
